# Multiples abcès froids tuberculeux associés à un mal de Pott chez un patient immunocompétent avec une localisation cervicale au Burkina Faso

**DOI:** 10.48327/mtsi.v5i1.2025.600

**Published:** 2025-01-06

**Authors:** Cheick Rachid BARGO, Wendbénédo Habacuc SARE, Mohamed Karfalla KABA, Modibo Abdoulaye NANA

**Affiliations:** 1 Service d’ORL et de chirurgie cervico-faciale du Centre hospitalier universitaire Bogodogo, Ouagadougou, Burkina Faso; 2 Unité d’ORL et de chirurgie cervico-faciale du Centre hospitalier universitaire Tengandogo, Ouagadougou, Burkina Faso; 3 Service d’ORL et de chirurgie cervico-faciale du Centre hospitalier universitaire Yalgado Ouédraogo, Ouagadougou, Burkina Faso; 4 Laboratoire d'analyse biomédicale du Centre hospitalier universitaire Tengandogo, Ouagadougou, Burkina Faso

**Keywords:** *Mycobacterium tuberculosis*, Mal de Pott, Antituberculeux, Abcès froid, Ouagadougou, Burkina Faso, Afrique subsaharienne, *Mycobacterium tuberculosis*, Pott's disease, Anti-tuberculosis drugs, Cold abscess, Ouagadougou, Burkina Faso, Sub-Saharan Africa

## Abstract

**Introduction:**

Le mal de Pott cervical est une localisation très rare de la tuberculose osseuse. Son association à des abcès froids cercico-thoraciques est également exceptionnelle.

**Observation:**

Patient de 36 ans, noir africain, d'origine burkinabè, avec notion de contage tuberculeux (mère atteinte de tuberculose pulmonaire), sans autre antécédent pathologique, reçu pour une cervicalgie chronique évoluant depuis six mois dans un contexte non fébrile et d'altération de l’état général. L'examen montrait des tuméfactions fluctuantes latéro-cervicale gauche et basithoracique gauche. L'examen pleuropulmonaire et neurologique était normal. La sérologie VIH et l'intradermoréaction à la tuberculine étaient négatives. La tomodensitométrie cervico thoracique a mis en évidence de multiples abcès. Il a été réalisé une incision drainage des abcès. L'analyse du prélèvement a montré la présence de bacille acido-alcoolo-résistant à l'examen direct, et de *Mycobacterium tuberculosis* au test Xpert avec une sensibilité à la rifampicine. Le diagnostic d'abcès froids tuberculeux multiples associé à un mal de Pott cervical a été posé. Le patient a été mis sous antalgique usuel et sous antituberculeux pendant 12 mois. L’évolution a été favorable avec un recul d'une année au-delà du traitement.

**Conclusion:**

L'abcès cervical froid compliquant un mal de Pott cervical est exceptionnel. L'imagerie et la technique d'étude geneXpert sont aujourd'hui des outils de diagnostic rapides et efficaces de la tuberculose. Cette forme particulière doit toujours être suspectée dans les zones endémiques.

## Abstract (en)

**Introduction:**

Cervical Pott's disease is a very rare localization of bone tuberculosis. Its association with cercico-thoracic cold abscesses is also exceptional.

**Observation:**

A 36-year-old black African patient of Burkinabè origin, with a history of tuberculosis infection (his mother had pulmonary tuberculosis) and no other pathological history, presented with chronic inflammatory cervicalgia that had been developing for six months in a non-febrile context of altered general condition. Examination showed a left latero-cervical swelling associated with a left basithoracic swelling, both fluctuant. Pleuropulmonary and neurological examinations were normal. HIV serology and tuberculin skin test were negative. Cervico-thoracic computed tomography revealed multiple abscesses. The abscesses were incised and drained. Analysis of the specimen showed the presence of acid-fast bacilli by direct examination and *Mycobacterium tuberculosis* by Xpert test, with sensitivity to rifampicine. The diagnosis was multiple tuberculous cold abscesses associated with cervical Pott's sore. The patient was started on standard analgesics and antituberculosis drugs for 12 months. The course was favorable, with a follow-up of one year after treatment.

**Conclusion:**

Cold cervical abscess complicating Pott's disease is exceptional. Imaging and the geneXpert assay are now rapid and effective diagnostic tools for tuberculosis. This particular form should always be suspected in endemic areas.

## Introduction

Le mal de Pott cervical ou sous occipital est défini par l'atteinte tuberculeuse des deux premières vertèbres cervicales ainsi que l'articulation occipito-atloïdienne et atloïdo-axoïdienne [15]. Le mal de Pott est une forme rare de tuberculose osseuse [13]. L'atteinte cervicale est exceptionnelle et elle représente moins de 3 % des cas de tuberculose vertébrale [9, 13]. Il peut être révélé par des suppurations rétro ou parapharyngées et la survenue d'abcès froids tuberculeux qui représentent une forme rare et inhabituelle de tuberculose extra pulmonaire [5, 8, 11, 14]. Par ailleurs, la localisation au niveau de la paroi thoracique est rare, et représente 0,1 % de toutes les localisations [2, 5]. Nous rapportons une présentation particulière de multiples abcès froids tuberculeux cervicothoraciques associant un mal de Pott cervical sans localisation pulmonaire primaire chez un sujet immunocompétent.

## Observation

**Figure 1:**
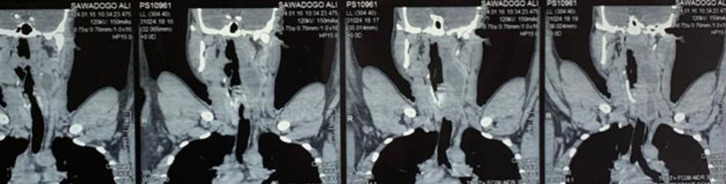
TDM cervico-thoracique (coupe frontale) mettant en évidence une collection hypodense à paroi rehaussée après injection de produit de contraste au niveau latéro pharyngolaryngé gauche

**Figure 2:**
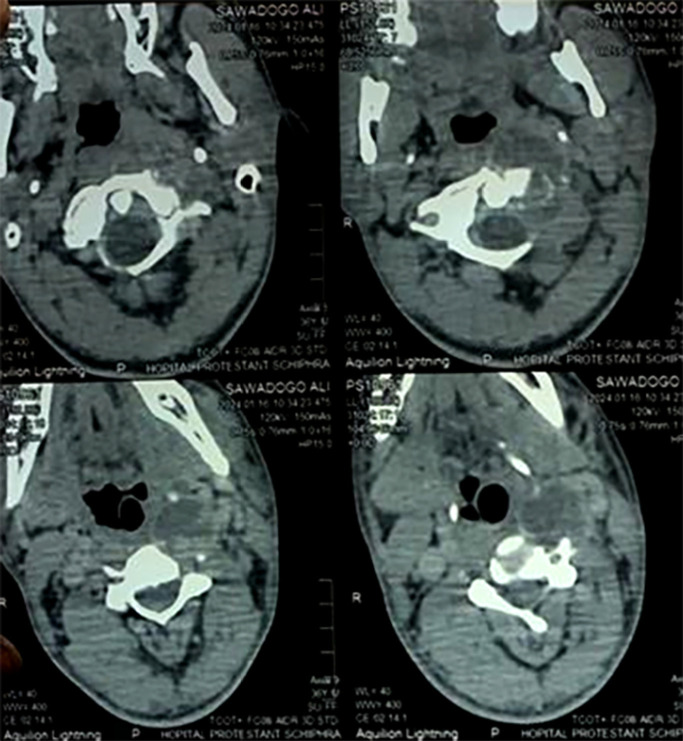
TDM cervicale (coupe axiale) passant par C1 mettant en évidence une collection hypodense rétropharyngée avec une lyse osseuse de l'arc antérieur de C1

**Figure 3:**
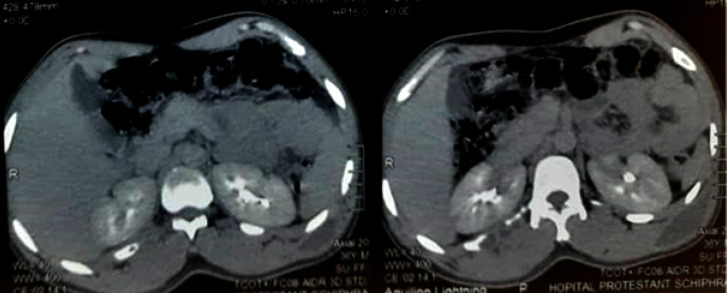
TDM thoraco-abdominale (coupe axiale) mettant en évidence deux collections thoraciques postéro inférieures bilatérales spontanément hypodenses et se rehaussant modérément après injection de produit de contraste

**Figure 4:**
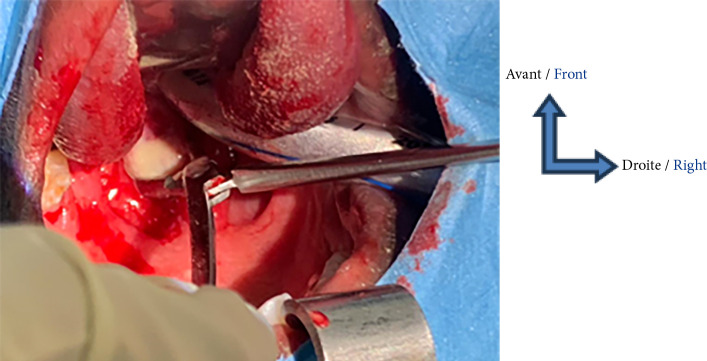
Vue opératoire de l'incision drainage par voie endobuccale de la collection retro et latéro pharyngolaryngée gauche

M. KI est un patient âgé de 36 ans, noir africain, d'origine burkinabé. Il est tabagique à cinq paquets-années. Il a une notion de contage tuberculeux, (mère atteinte de tuberculose pulmonaire), sans autre antécédent pathologique. Il consulte pour une cervicalgie chronique d'horaire inflammatoire associée à une raideur cervicale. Cette symptomatologie évoluerait depuis six mois dans un contexte apyrétique et d'altération de l’état général marquée par un amaigrissement (avec une perte pondérale chiffrée à 18 kg en 6 mois), une asthénie et une anorexie. Sa température était égale à 37,6 °C, un poids de 57 kg pour une taille 1,75 m (75 (indice de masse corporel 18,62 kg/m^2^). Le patient a déclaré avoir eu recours en premier lieu à la médecine traditionnelle. Le traitement traditionnel était constitué de décoctions à base d’écorces et de racines de plantes de nature non précisée. L'examen physique a mis en évidence au niveau cervical une tuméfaction latéro cervicale gauche ovalaire mesurant 10 cm de grand axe et 3 cm de petit axe, de consistance molle, indolore, fluctuante, avec une peau en regard saine, une douleur modérée à la palpation des épines cervicales. Au niveau thoracique, l'examen a retrouvé une tuméfaction en regard de l'arc postérieur de la 11^e^ et de la 12^e^ côte, mesurant 5 cm de grand axe, de consistance molle, indolore, fluctuante, avec une peau en regard saine. L'examen pleuropulmonaire et neurologique était normal. La sérologie VIH et l'intradermoréaction à la tuberculine étaient négatives. Le bilan rénal et hépatique était sans particularité. La tomodensitométrie (TDM) cervico thoracique a mis en évidence une collection liquidienne au niveau retro et latéro pharyngolaryngé gauche mesurant 141 × 32 × 27 mm avec une extension épidurale postérieure en regard de C1-C2 associée à une ostéolyse de l'arc antérieur de C1 et de la masse latérale gauche de C2 (Fig. 1, Fig. 2). Cette collection s’étend en bas jusqu'au niveau du plateau vertébral de T2 avec un refoulement d'une part du pharynx et du larynx à droite et, d'autre part, des gros vaisseaux du cou en dehors. A l’étage thoracique, la TDM a objectivé des collections liquidiennes bilatérales au niveau des muscles carrés des lombes, iliocostal droit et du muscle grand dorsal gauche, associées à une ostéite des arcs postérieurs des 11^e^ et 12^e^ côtes droites (Fig. 3). Des ganglions cervicaux bilatéraux ont aussi été retrouvés. Il a été réalisé une incision drainage sous anesthésie générale des collections par voie endobuccale pour les cervicales et par voie externe pour les thoraciques (Fig. 4). L'analyse du prélèvement a montré la présence de bacille acido-alcoolo-résistant à l'examen direct et de *Mycobacterium tuberculosis* au test Xpert avec une sensibilité à la rifampicine. Le patient a bénéficié d'une immobilisation cervicale à l'aide d'une minerve. Un traitement antituberculeux a été entrepris sur une période de 12 mois, avec deux mois de phase d'attaque associant quatre antituberculeux majeurs : rifampicine, isoniazide, éthambutol, pyrazinamide, suivis d'une phase d'entretien associant rifampicine et isoniazide pendant dix mois. L’évolution a été favorable avec une disparition complète de la symptomatologie et une amélioration de l’état général du patient avec un recul de 12 mois après le traitement.

## Discussion

La tuberculose demeure une infection endémique d'actualité. Elle pose un problème de santé publique particulièrement dans les pays en voie de développement [5, 8, 14]. La fréquence de la tuberculose extrapulmonaire varie dans le monde entier, représentant environ 15 à 30 % de tous les cas de tuberculose. Maiga *et al.* au Burkina Faso ont retrouvé une prévalence de 8,9 % de tuberculose extrapulmonaire [12]. L'insuffisance des moyens de dépistage disponibles au Burkina Faso pourrait expliquer la faible fréquence des formes extrapulmonaires.

Cette affection peut toucher divers organes, les ganglions lymphatiques et la plèvre étant les sites les plus fréquemment atteints [3, 4]. L'espace rétropharyngé, la colonne cervicale et la paroi thoracique sont des localisations peu courantes de la tuberculose extrapulmonaire [7]. Le mal de Pott est une forme rare de tuberculose osseuse et l'atteinte cervicale est exceptionnelle; elle représente moins de 3 % des cas de tuberculose vertébrale [9]. L'abcès rétropharyngé tuberculeux a une prévalence de 0,1 à 1 % parmi toutes les formes de tuberculose [16]. Par ailleurs, la localisation à la paroi thoracique des abcès froids tuberculeux est inhabituelle. Elle représente moins de 0,1 % de toutes les formes de tuberculose et 1 à 5 % des localisations ostéo-articulaires [6]. Ce cas est unique dans la mesure où le patient présentait une tuberculose extrapulmonaire impliquant simultanément trois localisations rares.

La pathogénie des abcès froids d'origine tuberculeuse n'est pas univoque. Les abcès froids se forment généralement à partir de foyers tuberculeux osseux ou ganglionnaires [5]. La bactérie provoque une réaction inflammatoire chronique, entraînant la nécrose des tissus infectés. Le pus s'accumule lentement sans provoquer de réaction inflammatoire aiguë. Cela explique l'absence de chaleur et de rougeur. Les abcès peuvent se propager aux tissus adjacents, créant des collections purulentes dans des zones comme la paroi thoracique, les muscles ou même les organes internes [5].

Les principaux diagnostics différentiels à évoquer lors de la découverte clinique d'abcès froid et/ou d'ostéite cervicale en milieu tropical sont l'abcès à pyogènes, à myobactéries atypiques, et les tumeurs du rachis cervical [8, 15]. Ces diagnostics partagent certains symptômes communs, notamment la douleur cervicale, la raideur cervicale et la faiblesse musculaire. Cependant, les abcès à pyogènes et à mycobactéries atypiques provoquent généralement une douleur aiguë et intense, tandis que les tumeurs du rachis cervical peuvent entraîner une douleur progressive au fil du temps. Par ailleurs, les abcès à pyogènes et à mycobactéries atypiques sont souvent accompagnés de signes inflammatoires tels que la fièvre, la rougeur et le gonflement, alors que les tumeurs du rachis cervical peuvent ne pas présenter ces signes inflammatoires.

Les abcès tuberculeux se présentent généralement sous la forme de lésions solitaires, bien que plusieurs sites puissent apparaître [5, 14]. De manière caractéristique, les abcès tuberculeux froids se manifestent par des signes non spécifiques, apparaissant souvent de manière insidieuse, et l'absence de fièvre dans des contextes suppuratifs chroniques devrait faire suspecter une tuberculose [5]. La confirmation du diagnostic repose sur l'isolement des mycobactéries à partir de liquide aspiré ou d’échantillons de biopsie, la technique GeneXpert facilitant le diagnostic rapide et la détection de la résistance, améliorant ainsi le début du traitement et le contrôle de la transmission [10, 14].

La prise en charge thérapeutique des abcès froids d'origine tuberculeuse associe chirurgie et traitement antituberculeux. Le geste chirurgical est à la fois diagnostique et thérapeutique, permettant le drainage complet de l'abcès et l'exérèse du tissu nécrotique sous-jacent, avec réalisation de biopsies pour obtenir une preuve histologique [1, 10, 14]. L'incision et le drainage ont été préférés à l'aspiration à l'aiguille dans notre cas, car le drainage et le débridement de l'abcès de la paroi thoracique pouvaient être effectués simultanément sous anesthésie générale. Après l'opération, notre patient a bénéficié d'une immobilisation cervicale à type de minerve. L’évolution a été favorable avec un recul d'une année au-delà du traitement. Le caractère multifocal des abcès tuberculeux chez notre patient peut s'expliquer par le retard de consultation dans un centre de santé. Ce retard est fréquent dans le contexte tropical africain. Les patients ont souvent recours en premier lieu aux tradipraticiens comme cela a été le cas pour notre patient. Ces retards peuvent altérer le pronostic. Cependant, le pronostic de notre patient a été favorable.

These delays may affect the prognosis. However, our patient's prognosis was good.

## Conclusion

Le mal de Pott cervical est exceptionnel. Il est caractérisé par une latence clinique et une symptomatologie diverse à l'origine d'un retard de prise en charge. Il s'agit d'une cause rare de suppuration cervicale. Il doit être évoqué essentiellement dans les pays d'endémie tuberculeuse. La prise en charge est médico-chirurgicale.

## Consentement du patient

Nous avons obtenu le consentement du patient pour la publication de cet article.

## Sources de financement

Les auteurs ne déclarent aucune source de financement.

## Contribution des auteurs

Cheick Rachid BARGO : rédaction, conception, discussion

Wendbénédo Habacuc SARE : conception, discussion

Karfalla Mohamed KABA, Abdoulaye Modibo

NANA : validation

## Conflit d'intérêt

Les auteurs ne rapportent aucun conflit d'intérêt.
