# Supplementary material for: Multiple tuberculous cold abscesses associated with Pott's disease in an immunocompetent patient with cervical localization in Burkina Faso
Source: Med Trop Sante Int. 2025 Jan 6;5(1):mtsi.v5i1.2025.629. [Article in French] doi: 10.48327/mtsi.v5i1.2025.600 (PMC12001987; doi:10.48327/mtsi.v5i1.2025.600)
Supplement: Related texts

# Research article: Multiple tuberculous cold abscesses associated with Pott's disease in an immunocompetent patient with cervical localization in Burkina Faso

## Introduction

Cervical or suboccipital Pott's disease is defined by tuberculous involvement of the first two cervical vertebrae and the occipito-atloid and atloid-axoid joints [15]. Pott's disease is a rare form of bone tuberculosis [13]. Cervical involvement is uncommon, accounting for less than 3% of vertebral TB cases [9, 13]. It may be manifested by retro- or parapharyngeal suppuration and the occurrence of cold tuberculous abscesses, which represent a rare and unusual form of extrapulmonary tuberculosis [5, 8, 11, 14]. Chest wall localization is rare, accounting for 0.1% of all localizations [2, 5]. We report a unique presentation of multiple cervicothoracic tuberculous cold abscesses associated with cervical Pott's disease without primary pulmonary localization in an immunocompetent subject.

## Observation

Mr. KI is a 36-year-old black African patient of Burkinabè origin. He smoked five pack-years. His mother had pulmonary tuberculosis, and he had no other pathological history. He presented with chronic inflammatory cervicalgia associated with cervical stiffness. This symptomatology had been evolving for six months, in the context of apyretic and altered general condition marked by weight loss (18 kg in 6 months), asthenia and anorexia. His temperature was 37.6°C, weight 57 kg and height 1.75 m (75 (BMI 18.62 kg/m^2^). The patient's first recourse was to traditional medicine. Traditional treatment consisted of bark and root decoctions of unspecified plants. Physical examination revealed an oval swelling on the left side of the cervix measuring 10 cm long and 3 cm short, soft, painless, fluctuating, with healthy skin and moderate pain on palpation of the cervical spines. On thoracic examination, a swelling was found opposite the posterior arch of the 11^th^ and 12^th^ ribs, measuring 5 cm long, soft, painless, fluctuating, with healthy skin. Pleuropulmonary and neurological examinations were normal. HIV serology and tuberculin skin test were negative. Renal and liver function tests were unremarkable. Cervico-thoracic computed tomography (CT) revealed a fluid collection in the left retro and latero-pharyngolaryngeal region measuring 141 × 32 × 27 mm, with posterior epidural extension opposite C1-C2, associated with osteolysis of the anterior arch of C1 and the left lateral mass of C2 (Fig. 1, Fig. 2). This collection extends down to the vertebral plateau of T2, with the pharynx and larynx on the right and the large vessels of the neck on the left. In the thoracic region, CT revealed bilateral fluid collections in the squared lumbar, right iliocostal and left latissimus dorsi muscles, associated with osteitis of the posterior arches of the 11^th^ and 12^th^ right ribs (Fig. 3). Bilateral cervical lymph nodes were also found. A drainage incision was made under general anaesthetic, using an endobuccal approach for the cervical lymph nodes and an external approach for the thoracic lymph nodes (Fig. 4).Analysis of the specimen showed the presence of acid-fast bacilli on direct examination and *Mycobacterium tuberculosis* on the Xpert test, with sensitivity to rifampin. The patient underwent cervical immobilization with a neck brace. Anti-tuberculosis treatment was initiated over a 12-month period, with a two-month attack phase combining four major anti-tuberculosis drugs: rifampicin, isoniazid, ethambutol and pyrazinamide, followed by a ten-month maintenance phase combining rifampicin and isoniazid. The course was favorable, with complete resolution of symptoms and improvement in the patient's general condition at 12 months post-treatment.

**Figure 1:** Cervicothoracic CT scan (frontal section) highlighting a hypodense collection with enhanced wall after injection of contrast product at the left lateropharyngolaryngeal level

**Figure 2:** Cervical CT scan (axial section) passing through C1 showing a retropharyngeal hypodense collection with bony lysis of the anterior arch of C1

**Figure 3:** Thoraco-abdominal CT scan (axial section) highlighting two bilateral postero-inferior thoracic collections that are spontaneously hypodense and moderately enhanced after injection of contrast agent

**Figure 4:** Operative view of the incision for intraoral drainage of the left retro and lateropharyngolaryngeal collection

## Discussion

Tuberculosis remains an endemic infection. It is a public health problem, especially in developing countries [5, 8, 14]. The incidence of extrapulmonary tuberculosis varies worldwide and accounts for approximately 15-30% of all tuberculosis cases. Maiga *et al.* found a prevalence of 8.9% for extrapulmonary TB in Burkina Faso [12]. The low frequency of extrapulmonary forms may be explained by the inadequacy of screening methods available in Burkina Faso.

The disease can affect various organs, with lymph nodes and pleura being the most common sites [3, 4]. The retropharyngeal space, cervical spine and chest wall are uncommon sites of extrapulmonary tuberculosis [7]. Pott's disease is a rare form of bone tuberculosis, and cervical involvement is exceptional, accounting for less than 3% of vertebral tuberculosis cases [9]. Tuberculous retropharyngeal abscesses have a prevalence of 0.1-1% among all forms of tuberculosis [16]. Chest wall localization of cold tuberculous abscesses is unusual. It accounts for less than 0.1% of all forms of tuberculosis and 1-5% of osteoarticular localizations [6]. This case is unique in that the patient presented with extrapulmonary tuberculosis in three rare localizations at the same time. The pathogenesis of cold abscesses of tuberculosis origin is not clear. Cold abscesses usually originate from tuberculous foci in bone or lymph nodes [5].

The bacterium causes a chronic inflammatory response that leads to necrosis of the infected tissue. Pus accumulates slowly without causing an acute inflammatory response. This explains the lack of heat and redness. Abscesses may spread to adjacent tissues, resulting in purulent collections in areas such as the chest wall, muscles, or even internal organs [5].

The main differential diagnoses to consider when cold abscesses and/or cervical osteitis are clinically discovered in tropical settings are pyogenic abscesses, atypical myobacterial abscesses, and cervical spine tumors [8, 15]. These diagnoses share certain common symptoms, particularly cervical pain, cervical stiffness, and muscle weakness. However, pyogenic and atypical mycobacterial abscesses usually cause acute, severe pain, while cervical tumors may cause progressive pain over time. On the other hand, pyogenic and atypical mycobacterial abscesses are often accompanied by inflammatory signs such as fever, redness, and swelling, whereas cervical tumors may not have these inflammatory signs.

Tuberculous abscesses usually present as solitary lesions, although multiple sites may occur [5, 14]. Characteristically, cold tuberculous abscesses present with nonspecific signs, often have an insidious onset, and the absence of fever in a chronic suppurative setting should raise suspicion for tuberculosis [5]. Confirmation of the diagnosis relies on isolation of mycobacteria from aspirated fluid or biopsy specimens, with the GeneXpert technique facilitating rapid diagnosis and detection of resistance, thereby improving treatment initiation and transmission control [10, 14].

The therapeutic management of cold abscesses of tuberculosis origin combines surgery and anti-tuberculosis treatment. The surgical procedure is both diagnostic and therapeutic, allowing complete drainage of the abscess and removal of the underlying necrotic tissue, with biopsies taken for histologic evidence [1, 10, 14]. Incision and drainage was preferred to needle aspiration in our case because drainage and debridement of the chest wall abscess could be performed simultaneously under general anesthesia. After surgery, our patient benefited from cervical immobilization with a cervical collar. The outcome was favorable with a one-year follow-up.

The multifocal nature of our patient's tuberculous abscesses may be explained by the delay in consulting a health center. This delay is common in tropical Africa. Patients often turn to traditional practitioners first, as was the case with our patient.

## Conclusion

Cervical Pott's disease is exceptional. It is characterized by clinical latency and diverse symptomatology, leading to delays in treatment. It is a rare cause of cervical suppuration. It should be considered especially in countries where tuberculosis is endemic. Management is medical-surgical.

## Patient consent

We have obtained the patient's consent for publication of this article.

## Funding

No funding was provided for this work.

## Authors’ contributions

Cheick Rachid BARGO: writing, design, discussion

Wendbénédo Habacuc SARE: conception, discussion

Karfalla Mohamed KABA, Abdo ulaye Modibo NANA: validation

## Conflict of interest

The authors declare no conflict of interests
